# Combined Metabolomics and Network Toxicology to Explore the Molecular Mechanism of *Phytolacca acinose* Roxb-Induced Hepatotoxicity in Zebrafish Larvae in Vivo

**DOI:** 10.1155/2021/3303014

**Published:** 2021-11-28

**Authors:** Dan Cao, Chongjun Zhao, Zhiqi Li, Qiqi Fan, Meilin Chen, Yangyu Jiang, Haiyan Wang, Hanjun Ning, Ruichao Lin, Jian Li

**Affiliations:** ^1^Beijing University of Chinese Medicine, Beijing 102488, China; ^2^Beijing Key Lab for Quality Evaluation of Chinese Materia Medica, Beijing 102488, China; ^3^Oncology Surgery Department, She Xian Hosipital, Handan 056400, China

## Abstract

*Phytolacca acinosa* Roxb (PAR), a traditional Chinese medicine, has been widely used as a diuretic drug for a long period of time for the treatment edema, swelling, and sores. However, it has been reported that PAR might induce hepatotoxicity, while the mechanisms of its toxic effect are still unclear. In this study, network toxicology and metabolomic technique were applied to explore PAR-induced hepatotoxicity on zebrafish larvae. We evaluated the effect of PAR on the ultrastructure and the function of the liver, predictive targets, and pathways in network toxicology, apoptosis of liver cells by PCR and western blot, and metabolic profile by GC-MS. PAR causes liver injury, abnormal liver function, and apoptosis in zebrafish. The level of arachidonic acid in endogenous metabolites treated with PAR was significantly increased, leading to oxidative stress in vivo. Excessive ROS further activated the p53 signal pathway and caspase family, which were obtained from KEGG enrichment analysis of network toxicology. The gene levels of *caspase-3*, *caspase-8*, and *caspase-9* were significantly increased by RT-PCR, and the level of Caps3 protein was also significantly up-regulated through western blot. PAR exposure results in the liver function abnormal amino acid metabolism disturbance and motivates hepatocyte apoptosis, furthermore leading to liver injury.

## 1. Introduction

*Phytolacca acinosa* Roxb (PAR), namely, *Shang Lu* in Chinese, has a long history in China, which is widely used as a diuretic drug in treating various diseases such as edema, nephritis, ascites, swelling, and sores [1]. PAR is recorded in Chinese Pharmacopoeia and is famous for its multiple functions, such as antibacterial, antiinflammatory, antiviral, antitumor, and for enhancing immunity by modern pharmacological study [2]. However, the toxic performance of PAR cannot be ignored, which may have a significant effect on target organ damage [3]. A study showed that a large dose of esculentoside A, a main active ingredient in PAR, has toxicity on HK-2 (human renal tubular epithelial cells) and the mechanisms of toxicity are associated with cellular oxidative damage and cell apoptosis [4]. Another study found that esculentoside A reduced the viability of HL7702 (human normal liver cells), causing hepatotoxicity [5]. However, until now, there have been few literatures on hepatotoxicity of PAR in vivo, and the mechanism of liver injury is still unclear.

Recently, zebrafish has been used to establish various disease models for high-throughput drug activity and toxicity screening [6–8], especially for a study on drug effects of the liver structure and function [9, 10]. At the molecular level, its hepatocellular function [11, 12] and histopathological changes in a variety of hepatic diseases are much similar to those of humans; therefore, zebrafish is an ideal experimental model for evaluating hepatotoxicity and metabolic research.

Metabonomic techniques could monitor the dynamic spectrum of endogenous metabolites [13, 14], quickly screen biomarkers with hepatotoxicity conditions, especially appropriate for the multipathway and multitarget toxicity complex system of TCM. Hepatotoxic drugs destroyed the homeostasis of endogenous substances by damaging the cell structure and functions. In addition, metabolomics is a considerable tool for tracing general metabolic changes in biological processes and investigating interactions of drug toxicity with biosomes.

Network toxicology belongs to the branch of the broad category of network pharmacology. By constructing a network model of interaction between toxicity-toxic component-toxic target-effect pathways and analyzing the correlation between specific components in the network toxicology [15] is extremely helpful to explore the toxicity mechanism on TCM.

In this study, for the sake of further clarification of the toxicity on PAR, we used zebrafish (*Danio* Rerio), a powerful vertebrate model organism, combined with comprehensive analysis of the function, morphology, and operation of hepatotoxicity, as well as metabonomics and network toxicology methods to explore the effects of zebrafish on liver injury and its potential toxicity mechanism.

## 2. Materials and Methods

### 2.1. Reagents and Materials

Methoxyamine HCl, fatty acid methyl ester (C7–C30, FAMEs) standards, pyridine, and anhydrous sodium sulfate were obtained from Sigma-Aldrich (St. Louis, MO, USA). MSTFA (N-methyl-N(trimethylsilyl)trifluoroacetamide) with 1% (vol/vol) trimethylchlorosilane (MSTFA, with 1% TMCS), methanol (Optima LC-MS), acetonitrile (Optima LC-MS), hexane, dichloromethane, chloroform, and acetone were purchased from Thermo-Fisher Scientific (FairLawn, NJ, USA). Ultrapure water was produced by a Mill-Q Reference system equipped with a LC-MS Pak filter (Millipore, Billerica, MA).

### 2.2. Toxicological Study of PAR-Induced Liver Injury

#### 2.2.1. Zebrafish Larvae

Wild-type (AB strain) zebrafish after the fertilization of 96hpf was provided by the Beijing key lab for quality evaluation by Chinese Materia Medica of Beijing University of Chinese Medicine. The incubation procedures and culture conditions of zebrafish eggs were referred to the published protocol [16] and approved by the Animal Ethics Committee of Beijing University of Chinese Medicine.

#### 2.2.2. Drug Exposure

*Phytolacca acinosa* Roxb (PAR) with batch number 200904 was provided by Anhui Guanghe Chinese Materia Medica Co., Ltd. (Anhui, China). PAR solutions with different solubility were prepared by double distilled water reflux extraction and stored at -20°C for determination.

#### 2.2.3. Lethality Curve

The zebrafish larvae from 96hpf after fertilization were randomly put into a 12-well plate with 20 zebrafish in each well. 4 mL of the PAR liquid was added to each well, and 3 replicates were set in the experiment. The 12-well plate was placed in the standard culture environment and kept at 28.5 ± 0.5°C for 14/10 h light-dark cycle. After PAR treatment for 24 hours, the death of zebrafish in each experimental group was observed and recorded under a DS-Qi2 fluorescence microscope (Nikon, Japan), and the average mortality of each group was calculated. SPSS20.0 was used to draw the best “mortality-concentration” effect curve, calculate the lethal concentration of the 10% zebrafish larvae (LC_10_), and used the LC_10_ concentration in the rest of the experiments to provide a reference for the concentration setting of the target organ identification test.

#### 2.2.4. Determination of ALT and AST

The zebrafish collected above were added to cold salt water (w/v = 1 : 9), homogenized in an ice bath, centrifuged (2500 rpm, 4°C) for 10 min, and the supernatant was taken according to the instructions provided in the detection kit (Nanjing Jiancheng Bioengineering Research Institute, Nanjing, China) for the determination of alanine aminotransferase (ALT) and aspartate aminotransferase (AST).

#### 2.2.5. Histopathological Evaluation of Hepatotoxicity

Zebrafish larvae were fixed in 4% paraformaldehyde for 72 hours and rinsed with running water, the tissues were soaked in different concentrations of ethanol gradually dehydrated, transparent in xylene and immersed with paraffin, and embedded in paraffin specimens at 65°C; the paraffin sections were cut by using a paraffin slicing machine (4 *μ*m/piece). After drying, hematoxylin and eosin (H&E) staining was used to observe the histopathological changes in zebrafish liver in the PAR-treated group and control group.

#### 2.2.6. Detection of Necrosis and Apoptosis of Hepatocytes

30 zebrafish larvae were randomly collected into an EP tube containing 1 mL dd H_2_O and 30 *μ*L 10 *μ*g/mL AO staining solution was added to avoid light staining for 1 h. The fluorescence in the zebrafish larvae were observed and photographed immediately by using a pose fluorescence microscope (Zeiss V16 Axio Zoom, 546 nm filter).

#### 2.2.7. Statistical Analysis

Statistical analysis was carried out by SPSS 20.0, and the data were expressed as mean ± standard deviation (*x* ± *s*). All data were accorded with normal distribution and the variance was homogeneous. The LC_10_ was calculated by one-way ANOVA and probability analysis. *P*-value<0.05 was considered to be statistically significant.

### 2.3. GC-MS Metabolomic Analysis

The sample preparation procedure was referred to the previously published method [6, 7]. The metabolites in the supernatant of liver homogenate were analyzed by the gas chromatography-time-of-flight-mass spectrometry (GC-TOF/MS) system, with Agilent 7890B gas chromatograph and a Gersted multipurpose sample MPS2 with dual heads (Muehlheim, Germany). A Rxi-5 MS capillary column (30m × 250 *μ*m, 0.25 *μ*m, Bellefonte, USA) was used for separation. Helium was used as the carrier gas at a constant flow rate of 1.0 mL/min. Initially, the instrument was maintained at a temperature of 80°C and was retained for 2 min. The temperature was increased to 300°C at a rate of 12°C/min and was retained at 300°C for 4.5 min. The temperature was increased from 300°C to 320°C at a rate of 40°C/min and was retained at 320°C for 1 min. The temperature of injection and the transfer interface were both set to 270°C. The source temperature was 220°C. The measurements were made by electron impact ionization (70 eV) in the full-scan mode (m/*z* 50–500). Instrument optimization was performed every 24 hours. 8 samples in each group were tested.

Nontargeted metabonomic analysis was carried out on the XploreMET platform (Metabo Profile, Shanghai, China). The biomarkers related to toxicity were screened and identified by principal component analysis (PCA) and orthogonal partial least square discriminant (OPLS-DA). The metabolic pathway enrichment and topological analysis of the differential metabolites were carried out by introducing MetaboAnalyst 4.0 to the study and analyzing the metabolic pathway of the biomarkers.

### 2.4. Network Toxicological Study

#### 2.4.1. Potential Target Prediction of Hepatotoxicity

The chemical constituents of PAR were retrieved from CNKI and PUBMED and imported into the phytochemical database of Traditional Chinese Medicine Systems Pharmacology Database (TCMSP, https://tcmspw.com/index.php/))) [17] to screen the putative targets of PAR. The Universal Protein Resource (UniProt) (https://www.uniprot.org/)) was used to select target protein names that transformed into Gene Symbol and standardized correction with the species defined as human.

Using “hepatotoxicity, liver toxicity, and liver injury” as key words, disease targets were searched in Genecards (http://www.genecards.org/)), Toxicogenomics Database (CTD, http://ctdbase.org/), and OMIM (https://omim.org/) databases [15, 18]. The data were cleaned and duplicated and were standardized by Uniprot database.

#### 2.4.2. Protein-Protein Interaction (PPI)

By using the online software mapping tool platform (http://www.bioinformatics.com.cn/login/), the component targets of PAR and liver toxicity related targets were mapped in Wayne diagram, and the intersection targets of PAR and hepatotoxicity were obtained, which were the key target of PAR and hepatotoxicity. The protein-protein interaction (PPI) network was constructed on the online platform of the STRING database (https://string- db.org/) [19]; the research species was defined as “*Homo sapiens*”, and the minimum interaction score was set to “medium confidence” (>0.4), while the other parameters were kept at the default setting.

#### 2.4.3. GO and KEGG Enrichment Analysis

The PAR-hepatotoxicity common genes were introduced into DAVID [20] database (https://david.ncifcrf.gov/), entered the target gene name list and set species defined as “*Homo* Sapiens”. GO analysis and KEGG [21] (Kyoto Encyclopedia of Genes and Genomes; https://www.kegg. Jp) pathway enrichment analysis was carried out, and enrichment results were visualized by the bioinformatics online tool to pick out the process and possible signal pathway of the PAR-induced liver injury.

### 2.5. Molecular Biological Verification

#### 2.5.1. QT-PCR Analysis

80 zebrafish larvae in each group were randomly selected and dissolved in Trizol reagent to extract total RNA. cDNA (complementary DNA) was synthesized by using a reverse transcription kit. TransStart Top Green and forward and reverse primers were used for real-time quantitative PCR (RT-PCR) analysis. The primer sequence is shown in Table 1. The experiment was conducted 3 times with different batches of zebrafish.

#### 2.5.2. Western Blot Test

Zebrafish whole fish (*n* = 80 per concentration) was homogenized on ice, and equal amounts of protein quantified by the BCA method (Beijing TDY Biotechnology Co., Ltd., WB0028) were separated by 12% SDS-PAGE, transferred to a nitrocellulose filter membrane (NC) membrane, and then blocked with 3%BSA-TBST. The primary antibody rabbit anti-caspase3 (ab13847, abcam) was incubated at 4°C overnight, followed by TBST washing and incubation with secondary antibody goat anti-rabbit IgG (*H* + *L*) HRP (Beijing TDY Biotech CO., Ltd. Beijing, S004) for 40 min. After washing for 1 min, it was reacted with ECL luminescent solution before exposure and was visualized by ChemiDoc XRS (Bio-Rad, Marnes-la-Coquette, France). The grayscale value of the exposure picture was analyzed by software ImageJ. The experiment was conducted 3 times with different batches of zebrafish.

## 3. Results

### 3.1. Toxicologic Study of PAR-Induced Liver Injury

#### 3.1.1. Lethal Curve

**Figure 1:**
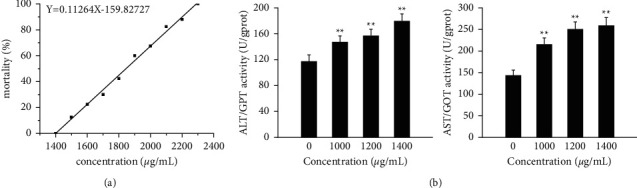
(a) Lethal curve from zebrafish induced by PAR from 96 hpf to 120 hpf. (b) The level of ALT and AST in zebrafish.

At the experimental exposure end point, the mortality rate was calculated by counting the number of zebrafish surviving in different PAR-treated groups, and the dose-toxicity curve of PAR-induced hepatotoxicity on zebrafish was obtained. Logistic regression analysis was carried out by SPSS20.0 statistical software, drawing the dose-toxicity curve, and calculating the LC_10_ values. Results of the acute toxicity experiments showed that the mortality of zebrafish larvae increased in a concentration-dependent manner with the increasing PAR exposure concentration. As shown in Figure 1(a), the LC_10_ value of PAR calculated from the lethal curve was 1503.69 *μ* g/mL.

#### 3.1.2. Liver Function Assessment

ALT and AST were amino acid transferases mainly distributed in hepatocytes, and they were commonly signal detection substances to evaluate liver injury through exogenous substances. When hepatocytes were injured, the structure of hepatocytes was destroyed, and their transaminases would be released into blood circulation, causing both content to be increased in plasma. In this study, compared with the control group, ALT and AST values of zebrafish larvae exposed to PAR increased significantly as the concentration increased (Figure 1(b)). It is noteworthy that ALT activity was more sensitive to drug exposure, and the results were consistent with rodents and mammals, which indicated that PAR induced liver injury in zebrafish.

#### 3.1.3. Histopathological Evaluation of Hepatotoxicity in Zebrafish

**Figure 2:**
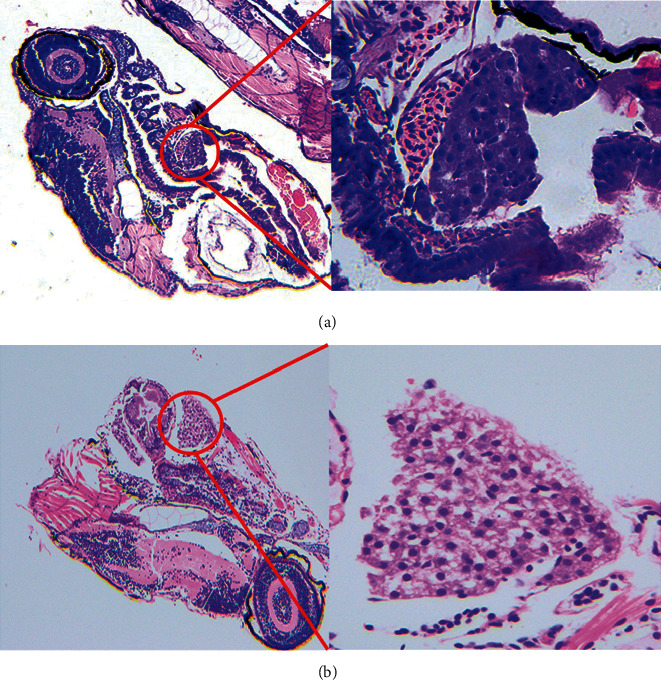
Histopathology of zebrafish liver in both control group and PAR-treated group. (a) Control (100×, 400×). (b) PAR (100×, 4 00×).

We studied the histopathological characteristics of zebrafish to evaluate the effect of PAR on hepatotoxicity. In the control group, hepatocytes were normal in the sections of zebrafish larvae with intact liver cells and were tightly arranged regularly (Figure 2(a)). In the PAR treatment group, the liver size and optical density of most zebrafish larvae changed significantly, the number of hepatocytes was reduced, and vacuolar degeneration and irregular arrangement appeared, which were consistent with the traditional endpoint time in published articles. PAR-treated liver tissue cells exhibited obvious morphological changes and local necrosis, which showed PAR-induced liver injury (Figure 2(b)).

#### 3.1.4. In Vivo Cell Death Assay Results

**Figure 3:**
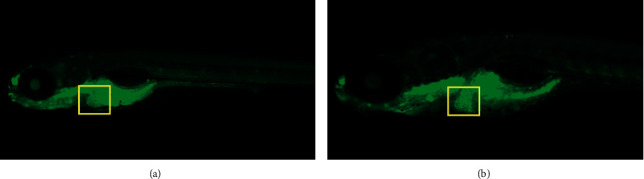
In vivo cell death and apoptosis results. (a) Control group. (b) PAR group.

AO staining displayed hepatocyte necrosis and apoptosis of zebrafish larvae, while the control group and the PAR-treated group showed typical whole cell death images. The results revealed that the PAR-treated larvae had a significant inhibitory effect on apoptosis (Figure 3).

### 3.2. Metabolome Analysis

**Figure 4:**
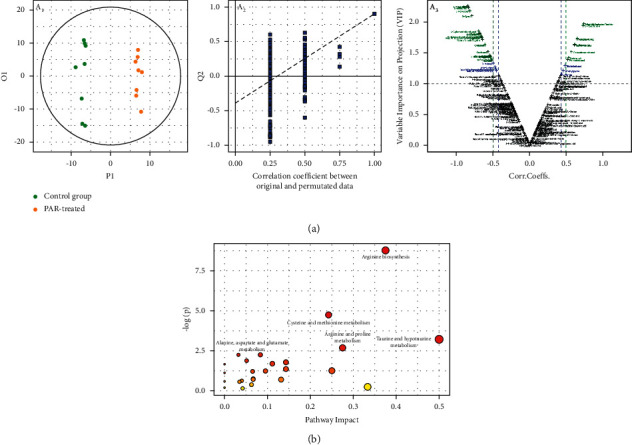
(a) Visualization of the overall metabolite profile difference between the PAR-treated group and the control group including the OPLS-DA predictive/discriminate score plot (a_1_), 1000 permutation tests (a_2_), and V-plot (a_3_); (b) differential metabolic pathways between the PAR-treated group and the control group.

The unsupervised pattern recognition method, PCA analysis, can reflect the original state of the data and directly show the overall differences between different samples, so samples in the control group and PAR-treated group were analyzed by PCA. It can be seen from Figure 4(a) that the samples of the two groups were significantly separated, indicating that there are some metabolic differences between the two groups.

With a significance level of 0.05, a corresponding Corr. Value was used as a cutoff value to select the variables that were most correlated with the very first predictive components (Figure 4(a)). The diagnostic parameters of the PLS-DA model were summarized in Figure 4(a). Each point in the volcano chart represented a variable, and the importance of the variable in the classification was measured by the value of VIP (variable importance in the projection). The farther the variable was from the origin, the greater the VIP value.

The variables were screened according to the VIP value and *p*-value, and VIP > 1 and *p* < 0.05 were used as candidates for seeking differential metabolites. As shown in Table 2, 35 different metabolites were screened in this study. Compared with the control group, 9 substances were up-regulated (red in Table 2) and 26 substances were down-regulated (green in Table 2) in the samples of the administration group, which might suggest that these 35 endogenous metabolites were related to the liver function damage of zebrafish induced by PAR. Based on the KEGG enrichment analysis, arginine biosynthesis, arginine and proline metabolism (Figure 4(b)), taurine and hypotaurine metabolism, and cysteine and methionine metabolism were selected as potential pathways by using the MetaboAnalyst 3.0 online system (MetPA, https://www.metaboanalyst.ca/).

### 3.3. Network Toxicological Study

#### 3.3.1. Potential Target Prediction of Hepatotoxicity

**Figure 5:**
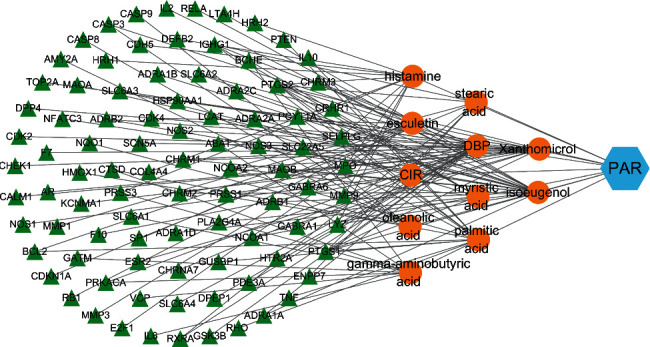
Network construction of the “PAR-component-target (note: the orange nodes represent the PAR component and green nodes represent the targets of the PAR component).

The chemical constituents of PAR retrieved from CNKI and PUBMED databases were all imported into TCMSP database for retrieval. 11 compounds with OB ≥ 30% and DL ≥ 0.18 were screened out according to oral bioavailability and drug-like analysis, and a total of 92 targets were obtained in the TCMSP database. The PAR component-target network consists of 92 target nodes and 11 active component nodes (Figure 5).

All the hepatotoxic related targets were searched through Genecards, TCD, and OMIM, the duplicates were deleted and 8476 target proteins were integrated, and all the predicted target proteins were converted into corresponding gene names through Uniprot database.

#### 3.3.2. Protein-Protein Interaction (PPI)

**Figure 6:**
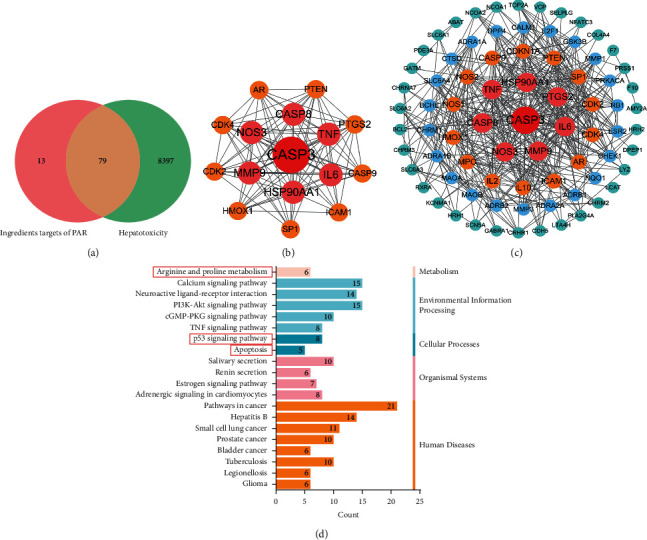
Analysis results of network toxicology. (a) Venn diagram of 79 potential targets of PAR, which were intersected by “ingredient targets” and “toxic targets”. (b) PPI analysis of core targets from PAR. (c) PPI analysis of common targets of PAR. (d) KEGG pathway analysis by David database.

To further explore the mechanism of hepatotoxicity induced by PAR, we found that 79 targets were overlapped in both PAR active components and hepatotoxic targets (Figure 6(a)). These 79 targets were imported into STRING database to analyze the relationship between proteins, and the PPI network was obtained (Figure 6(c)). In the network, nodes represent targets, edges represent the interaction between targets, and different colors represent different interactions. The PPI network was analyzed by Cytoscape3.7.2, the degree value median was 9.5, and the average topology coefficient was 0.350. According to the degree values, 16 core targets with degree >19 were screened, namely, CASP3, TNF, IL6, MMP9, NOS3, HSP90AA1, CASP8, PTGS2, AR, SP1, PTEN, CDK2, ICAM1, CDK4, CASP9, and HMOX1; for specific information, see Figure 6(b). These may be potential targets for PAR-induced hepatotoxicity.

#### 3.3.3. GO and KEGG Enrichment Analysis

The DAVID database was used to analyze the functional enrichment of 78 PPI targets. In the GO functional enrichment analysis, the first 10 items of the biological process, cellular composition, and molecular function were selected (Table 3). The biological process involved negative regulation of calcium ion transport, positive regulation of vasoconstriction, and negative regulation of apoptotic process; the cellular component involves caveola, membrane raft, asymmetric synapse etc.; molecular function mainly involves protein homodimerization activity, nitric-oxide synthase activity, norepinephrine binding, etc.

In the KEGG pathway enrichment analysis, the first 20 items were selected to draw a bar chart (Figure 6(d)). The main pathways related to hepatotoxicity are metabolic pathway, namely, arginine and proline metabolism; PI3K-Akt signaling pathway in environmental information procession; p53 signaling pathway and TNF signaling pathway in cellular processes; and apoptosis pathway. Based on the above results of cyber toxicological studies on these core targets, biological processes, and signal pathways on PAR-induced hepatotoxicity, it was found that the screened out active components of PAR with core targets exerted hepatotoxicity effects that were related to a variety of biological processes, whose mechanisms may be closely related to the regulation of physiological processes such as apoptosis, cell proliferation, and oxidative stress.

### 3.4. Molecular Biology Verification

#### 3.4.1. QT-PCR Analysis of Key Target Genes

**Figure 7:**
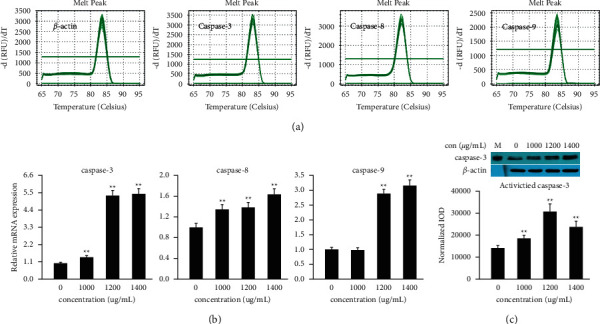
(a, b) Gene expression of *caspase-3*, *caspase-8*, and *caspase-9* was examined by QT-PCR in groups treated with PAR. (c) Protein expression of activated Caps3 was examined by western blot in the zebrafish larvae group treated with PAR, *n* = 80. ^*∗*^indicates *p* < 0.05 versus the control group, ^*∗∗*^indicates *p* < 0.01 versus the control group, by one-way ANOVA.

The P53 signal pathway and apoptosis signal pathway are located on the same signal axis. Because the apoptosis signal pathway was a key factor in tissue injury, and the caspase family played an important role in the regulation of apoptosis, we used different concentrations of PAR to induce zebrafish to detect the transcriptional levels of *caspase-3*, *caspase-8*, and *caspase-9*. As shown in Figure 7(a), compared with the normal control group, the PAR of 1000–1400 *μ*g/mL had significant effects on *caspase3*, *caspase-8*, and *caspase-9* genes of zebrafish. The expression levels of *caspase-3* and *caspase-8* were significantly increased at 1000–1400 *μ*g/mL, while the expression of *caspase-9* was significantly increased when the concentration of PAR was 1200–1400 *μ*g/mL. The mentioned results revealed that hepatocyte apoptosis and hepatotoxicity occurred in zebrafish hepatocytes within 4 dpf after the administration of PAR.

#### 3.4.2. Western Blot Analysis of Caps3 Protein

The molecular mechanism of apoptosis according to whether it depended on Caspase or not can be divided into two types, Caspase-dependent and Caspase-independent. The former is the most classical apoptosis pathway, and the vast majority of cells induce apoptosis by activating Caspase. Among the Caspase family proteins, Caps3 was the downstream of the apoptosis regulation pathway, which was the key executive molecule in the apoptosis signal transduction pathway, so it is also called the death protease. To further detect the expression level of protein Caps3, we used western blot to detect the protein expression of Caps3 in zebrafish. As shown in Figure 7(b), the expression of Caps3 in the liver of zebrafish treated with PAR was significantly up-regulated, indicating that PAR induced hepatotoxicity in zebrafish in a Caspase-dependent manner by activating Caps3.

## 4. Discussion

Over the course of the past decades, there has been a worldwide effort aimed at developing drugs from natural products to treat various health conditions and tend to make the TCM as potential therapeutic options of worldwide importance. The reasons for this phenomenon may be related to a belief that herbal TCMs labeled as “natural” are always safe and of benefit to public health, improving physical fitness, extending lifespan, and treating various illnesses. Recently, the abundance of the reported liver injury cases induced by the TCM has attracted wide public and regulatory attention, especially related to hepatotoxicity, which can evolve into acute liver failure in most serious cases [22, 23]. It is known that hepatotoxicity results from a sequence of induced mechanisms and is involved with different liver toxic phenotypes, such as cholestasis [24], steatosis [25], and necrosis because of different external factors, which brings great difficulties in the definitive diagnosis of most TCM-induced hepatotoxicity in the clinic and drug development.

In this study, we used zebrafish as an animal model [26–28] to observe the liver morphology of the zebrafish induced by PAR, the changes of biochemical indexes of liver function enzymes, and the effect of AO staining on hepatotoxicity. Combined with nontargeted metabolites by using GC-MS, network toxicology and molecular biology methods were used to find out differential metabolites and metabolic pathways to elucidate the potential mechanism of zebrafish hepatotoxicity induced by PAR.

To evaluate the hepatotoxicity of PAR, we performed tissue sections and AO staining on whole zebrafish to determine whether PAR could destroy the liver. At the same time, acute toxicity experiments were carried out to observe the lethality of zebrafish treated with different PAR concentrations. Compared with the control group, the PAR-treated group exhibited green fluorescence, which indicated that hepatocytes were obviously apoptotic after AO staining. We found some changes in hepatocytes including the decrease in number, vacuolar degeneration, irregular arrangement, destruction of cell structure, and even degeneration. As ALT and AST are the common indicators for the clinical evaluation of hepatotoxicity, ALT and AST levels in the PAR-exposure group were significantly increased than those in the control group (*p* < 0.05). Therefore, ALT and AST accumulated in the zebrafish liver by the induction of PAR, resulting in hepatocyte damage and necrosis.

Nontargeted metabolomics were applied for seeking different metabolites and probable metabolic pathways. In this study, we found that the metabolic pathways arginine biosynthesis, arginine and proline metabolism, taurine and hypotaurine metabolism, cysteine and methionine metabolism were perturbed by PAR. Methionine protected liver mainly related to the following two aspects: (1) promoting phospholipid methylation of hepatocyte membrane, increasing membrane fluidity, and reducing cholestasis in hepatocytes and (2) strengthening the trans-sulfuration function, so as to accelerate the synthesis of cysteine, glutathione, and taurine in hepatocytes, and improve the antioxidant capacity of organism [29, 30]. Methionine can induce endogenous antioxidant response by activating the Nrf2-ARE pathway, while in turn improving the ROS-derived oxidative stress. In this study, both cysteine and methionine on PAR-treated zebrafish liver were decreased significantly, which might inhibit the sulfur transfer pathway of methionine, causing abnormal metabolism of cysteine and methionine, and leading to redox imbalance; therefore, hepatocytes were more vulnerable to attack and damage by free radicals.

The metabolism of proline was down-regulated, indicating that the arginine and proline metabolism was disturbed. Proline is a nonessential amino acid, mainly synthesized by glutamate, which plays important roles in carbon and nitrogen metabolism, oxidative stress protection [31], protein synthesis, and programmed cell death [32]. Urea is produced mainly in liver, the level of urea metabolism decreased, reflecting a perturbation of purine metabolism. In purine metabolism, hypoxanthine is oxidized by xanthine oxidase (XOD) to produce uric acid and excessive oxygen-free radicals, while excessive oxygen free radicals lead to ROS accumulation and oxidative stress [33]. In this study, hypoxanthine decreased and isopterin increased, suggesting that hypoxanthine and isopterin participated in to be a harmful outcome of PAR-induced hepatotoxicity, disordered purine nucleotide metabolism, and triggered oxidative stress response in zebrafish in vivo.

Arachidonic acid can produce a large amount of reactive oxygen species through metabolic enzymes such as cyclooxygenase (COX) and lipoxygenase (LOX) to aggravate oxidative stress and induce oxidative stress in hepatocytes [34]. Increasing the arachidonic acid could increase the production of ROS. While excessive ROS may attack intracellular lipids and initiate a series of signal cascade reactions that affect apoptosis, related gene and protein active product expression, biofilm lipid peroxidation, and tissue damage can at last give rise to liver injury. In this study, the level of arachidonic acid of PAR was increased, releasing a large amount of reactive oxygen species, aggravating oxidative stress, ultimately destroying the balance of redox and organism damage.

We also studied the network toxicology of PAR and screened *caspase-3*, *caspase-8*, and *caspase-9* as potential core targets. As we know, the vast majority of cells induce apoptosis by activating the Caspase family, predicting that the p53 signal pathway and arginine and proline metabolism are key pathways to induce liver injury. In this experiment, zebrafish treated with PAR generated significant responses at *caspase-3*, *caspase-8*, and *caspase-9* gene levels. In addition, activated Caps3 was evaluated following toxic exposure at a sublethal concentration. Caps3 is not only the most important terminal cleavage enzyme that caused apoptosis, but also has an irreplaceable role executing the molecule of apoptosis. These results confirmed hepatocyte apoptosis occurred after zebrafish was treated with PAR, and *caspase-3*, *caspase-8*, and *caspase-9* were significantly up-regulated, which also verified the prediction via network toxicology.

**Figure 8:**
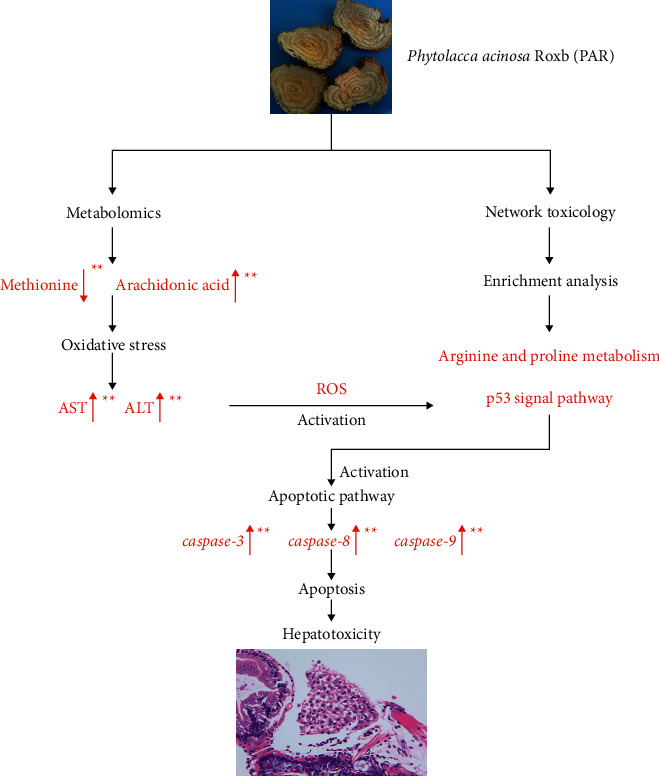
Molecular mechanism of PAR-induced hepatotoxicity.

Our results demonstrate a strong correlation between PAR and liver injury. Compared with the control group, the liver tissue of zebrafish in the PAR exposure group had pathological changes, and the liver function test suggested that PAR could cause hepatotoxicity. The metabolic disorder of amino acid was found to up-regulate arachidonic acid through metabonomics, and network toxicology was used to screen the key signal pathways (p53 signal pathway and arginine and proline metabolism). High levels of arachidonic acid and descendant methionine can trigger oxidative stress and excessive ROS, which may activate the p53 pathway and cause apoptosis. The overexpression of *caspase-3*, *caspase-8*, and *caspase-9* can induce hepatocyte apoptosis and eventually resulted in liver injury (Figure 8).

## 5. Conclusion

The molecular mechanism of hepatotoxicity induced by PAR was analyzed by nontargeted metabolomics, molecular biology, and network toxicology. First, a comprehensive analysis of liver alterations, including liver morphology and function of zebrafish liver was observed. Subsequently, the changes in endogenous metabolites, especially amino acids analysis and network toxicology, were predicted. Finally, the genes and proteins related to liver injury were verified. Besides, this study provides the basis for the use of zebrafish in the prediction of drug hepatotoxicity and reference for future studies on the hepatotoxicity mechanism in other models and supply data for its safety on clinical use.

## Figures and Tables

**Table 1:** The sequences of the primers.

Target gene	Accession ID	Forward primer sequence (5′-3′)	Reverse primer sequence (5′-3′)
*β*-actin	AF057040.1	GGCTGTGCTGTCCCTGTAT	GGGCGTAACCCTCGTAGAT
Caspase-3	AB047003.1	TCAGGCTTGTCGAGGAAC	CTGCCATACTTTGTCATCATTT
Caspase-8	NM_131510.2	AAGACCTGATTCTGCGACTG	TAGGCTGAGACACCTTTACG
Caspase-9	NM_001007404.2	TTCATCGCCCTCCTGTC	CTGGCATCCATCTTGTAGC

**Table 2:** 35 metabolites contributing to the separation between the control and treated groups exposed to PAR (*p* < 0.05).

Class	Metabolites	Formula	HMDBID	KEGGID	VIP	*P*-value	FC	Corr.Coeffs.	Effect
Amino acid	Urea	CH_4_N_2_O	HMDB00294	C00086	2.2	6.49*E* − 04	0.6	−0.84	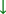
Amino acid	Ornithine	C_5_H_12_N_2_O_2_	HMDB00214	C00077	2.2	7.21*E* − 05	0.7	−0.84	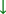
Amino acid	L-Tryptophan	C_11_H_12_N_2_O_2_	HMDB00929	C00078	2.2	3.08*E* − 04	0.8	−0.84	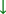
Amino acid	L-Methionine	C_5_H_11_NO_2_S	HMDB00696	C00073	2.2	1.56*E* − 04	0.8	−0.83	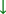
Amino acid	L-norleucine	C_6_H_13_NO_2_	HMDB01645	C01933	2.2	2.95*E* − 04	0.7	−0.82	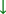
Carbohydrates	D-Xylose	C_5_H_10_O_5_	HMDB00098	C00181	2.1	7.02*E* − 04	0.6	−0.79	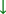
Nucleotide	Isoxanthopterin	C_6_H_5_N_5_O_2_	HMDB00704	C03975	2.0	1.25*E* − 03	1.7	0.74	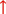
Amino acid	Ratio of putrescine/ornithine	C_4_H_12_N_2_/C_5_H_12_N_2_O_2_	HMDB01414/HMDB00214	C00134/C00077	2.0	1.71*E* − 03	1.1	0.74	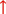
Organic acids	L-Pipecolic acid	C_6_H_11_NO_2_	HMDB00716	C00408	1.9	2.52*E* − 03	2.5	0.73	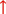
Amino acid	L-Alpha-aminobutyric acid	C_4_H_9_NO_2_	HMDB00452	C02356	1.8	4.70*E* − 03	0.8	−0.69	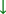
Amino acid	L-Cystine	C_6_H_12_N_2_O_4_S_2_	HMDB00192	C00491	1.8	3.92*E* − 03	0.7	−0.68	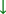
Amino acid	L-Threonine	C_4_H_9_NO_3_	HMDB00167	C00188	1.8	8.37*E* − 03	0.8	−0.68	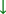
Nucleotide	Hypoxanthine	C_5_H_4_N_4_O	HMDB00157	C00262	1.8	5.34*E* − 03	0.9	−0.67	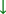
Amino acid	L-Cysteine	C_3_H_7_NO_2_S	HMDB00574	C00097	1.8	1.10*E* − 02	0.8	−0.66	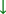
Organic acids	2-Hydroxy-3-methylbutyric acid	C_5_H_10_O_3_	HMDB00407	NA	1.8	7.05*E* − 03	0.8	−0.66	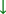
Amino acid	2-Aminoadipate	C_6_H_11_NO_4_	HMDB00510	C00956	1.7	8.61*E* − 03	0.8	−0.65	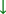
Nucleotide	Ratio of hypoxanthine/inosinic acid	C_5_H_4_N_4_O/C_10_H_13_N_4_O_8_P	HMDB00157/HMDB00175	C00262/C00130	1.7	5.96*E* − 03	0.7	−0.65	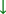
Amino acid	L-Asparagine	C_4_H_8_N_2_O_3_	HMDB00168	C00152	1.7	9.63*E* − 03	0.6	−0.65	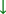
Amino acid	Methylcysteine	C_4_H_9_NO_2_S	HMDB02108	NA	1.7	1.38*E* − 02	2	0.65	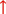
Vitamin	Pantothenic acid	C_9_H_17_NO_5_	HMDB00210	C00864	1.7	6.53*E* − 03	1.4	0.65	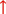
Amino acid	Ratio of urea/L-arginine	CH_4_N_2_O/C_6_H_14_N_4_O_2_	HMDB00294/HMDB00517	C00086/C00062	1.7	9.49*E* − 03	0.7	−0.64	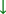
Alkylamines	Putrescine	C_4_H_12_N_2_	HMDB01414	C00134	1.6	0.0159	0.8	−0.61	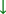
Amino acid	L-Homoserine	C_4_H_9_NO_3_	HMDB00719	C00263	1.6	0.0171	2.3	0.61	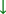
Amino acid	L-Proline	C_5_H_9_NO_2_	HMDB00162	C00148	1.6	0.0237	0.9	−0.61	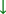
Fatty acids	Arachidonic acid	C_20_H_32_O_2_	HMDB01043	C00219	1.6	0.0113	1.4	0.61	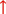
Fatty acids	Docosahexaenoic acid	C_22_H_32_O_2_	HMDB02183	C06429	1.5	0.0248	1.3	0.57	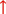
Organic acids	Quinic acid	C_7_H_12_O_6_	HMDB03072	C06746	1.5	0.0238	0.4	−0.57	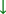
Carbohydrates	Alpha-lactose	C_12_H_22_O_11_	HMDB00186	C00243	1.4	0.0432	0.7	−0.54	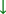
Lipids	MG160	C_19_H_38_O_4_	HMDB11564	NA	1.4	0.0353	0.9	−0.54	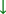
NA	3-Hydroxypyridine	C_5_H_5_NO	NA	NA	1.4	0.0359	0.9	−0.54	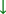
Carbohydrates	L-Arabitol	C_5_H_12_O_5_	HMDB01851	C00532	1.4	0.0344	0.9	−0.54	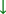
Fatty acids	Myristic acid	C_14_H_28_O_2_	HMDB00806	C06424	1.4	0.0405	1.2	0.53	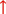
Amino acid	Homocysteine	C_4_H_9_NO_2_S	HMDB00742	C00155	1.4	0.0367	0.8	−0.52	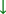
Organic acids	Petroselinic acid	C_18_H_34_O_2_	HMDB02080	C08363	1.4	0.0334	1.4	0.52	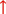
Lipids	MG182	C_21_H_38_O_4_	HMDB11568	NA	1.4	0.0477	0.7	-0.51	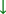

**Table 3:** GO analysis of potential targets.

GO ID	Terms	Subgroup	Gene counts	P-value
GO:0042493	Response to drug	Biological process	18	3.58 × 10^−14^
GO:0032355	Response to estradiol	Biological process	10	3.61 × 10^−10^
GO:0001666	Response to hypoxia	Biological process	10	1.00 × 10^−7^
GO:0051926	Negative regulation of calcium ion transport	Biological process	5	2.98 × 10^−7^
GO:0045907	Positive regulation of vasoconstriction	Biological process	6	3.47 × 10^−7^
GO:0051384	Response to glucocorticoid	Biological process	7	5.49 × 10^−19^
GO:0045471	Response to ethanol	Biological process	8	5.61 × 10^−18^
GO:0035094	Response to nicotine	Biological process	6	7.37 × 10^−7^
GO:0007568	Aging	Biological process	9	9.59 × 10^−7^
GO:0043066	Negative regulation of apoptotic process	Biological process	13	1.16 × 10^−6^
GO:0045121	Membrane raft	Cellular component	10	2.35 × 10^−7^
GO:0043005	Neuron projection	Cellular component	10	1.18 × 10^−6^
GO:0005886	Plasma membrane	Cellular component	38	5.79 × 10^−6^
GO:0005887	Integral component of plasma membrane	Cellular component	20	8.52 × 10^−6^
GO:0005901	Caveola	Cellular component	6	3.60 × 10^−5^
GO:0005576	Extracellular region	Cellular component	20	3.60 × 10^−5^
GO:0016324	Apical plasma membrane	Cellular component	8	2.47 × 10^−4^
GO:0032279	Asymmetric synapse	Cellular component	3	4.98 × 10^−4^
GO:0005615	Extracellular space	Cellular component	16	5.18 × 10^−4^
GO:0000307	Cyclin-dependent protein kinase holoenzyme complex	Cellular component	3	0.00183
GO:0019899	Enzyme binding	Molecular function	10	2.24 × 10^−5^
GO:0008144	Drug binding	Molecular function	6	2.64 × 10^−5^
GO:0042803	Protein homodimerization activity	Molecular function	14	2.73 × 10^−5^
GO:0004517	Nitric-oxide synthase activity	Molecular function	3	6.30 × 10^−5^
GO:0051380	Norepinephrine binding	Molecular function	3	1.26 × 10^−4^
GO:0034617	Tetrahydrobiopterin binding	Molecular function	3	1.26 × 10^−4^
GO:0004252	Serine-type endopeptidase activity	Molecular function	8	1.75 × 10^−4^
GO:0051379	Epinephrine binding	Molecular function	3	3.12 × 10^−4^
GO:0031625	Ubiquitin protein ligase binding	Molecular function	8	3.60 × 10^−4^
GO:0020037	Heme binding	Molecular function	6	4.30 × 10^−4^

## Data Availability

The data used to support the findings of this study are included within the article.

## Abbreviations

TCM: Traditional Chinese Medicine
PAR: Phytolacca acinosa Roxb
HK-2: Hexokinase 2
ALT: Alanine transaminase
AST: Aspartate transaminase
PBS: Phosphate buffer saline
H&E: Hematoxylin and eosin
AO: Acridine orange
GC-MS: Gas chromatography-mass spectrometer
PCA: Principal component analysis
PLS-DA: Partial least squares discriminant analysis
KEGG: Kyoto Encyclopedia of Genes and Genomes
DAVID: Database for Annotation Visualization and Integrated Discovery
TCMSP: Traditional Chinese Medicine Systems Pharmacology
CNKI: Chinese National Knowledge Infrastructure
UniProt: Universal Protein Resource
GO: Gene Ontology
PPI: Protein-Protein Interaction
CC: Cellular Component
BP: Biological Process
MF: Molecular Function
FC: Fold changes
VIP: Variable importance in projection
XOD: Xanthine oxidase
COX: Cyclooxygenase
LOX: Lipoxygenase.
